# Gualou Guizhi Granule Protects against OGD/R-Induced Injury by Inhibiting Cell Pyroptosis via the PI3K/Akt Signaling Pathway

**DOI:** 10.1155/2021/6613572

**Published:** 2021-03-08

**Authors:** Yuqin Zhang, Hongyun Wang, Huang Li, Lihong Nan, Wei Xu, Yu Lin, Kedan Chu

**Affiliations:** ^1^Pharmacy College, Fujian University of Traditional Chinese Medicine, Fuzhou, Fujian, China; ^2^State Key Laboratory of Chinese Pharmacies, Fujian Provincial Department of Science and Technology, Fujian University of Traditional Chinese Medicine, Fuzhou, Fujian, China

## Abstract

Pyroptosis is a proinflammatory form of regulated cell death that plays an important role in ischemic stroke. Gualou Guizhi granule (GLGZG) is a classic prescription that has been shown to exert neuroprotective effects against cerebral ischemia reperfusion injury. In the present study, we examined the involvement of pyroptosis and its associated mechanism in protecting nerve function. *Methods*. Primary neurons were exposed to oxygen-glucose deprivation and reperfusion (OGD/R) conditions in the presence or absence of GLGZG. Cellular viability was measured by the 3-(4,5-dimethylthiazol-2-yl)-2, 5-diphenyltetrazoliumbromide (MTT) assay. The number of apoptoic cells was detected by NeuN and NSE protein expression. The expression levels of the pyroptosis markers, namely, NOD-like receptor family pyrin domain-containing 3 (NLRP3), apoptosis-associated speck-like protein containing a CARD (ASC), caspase-1, interleukin-18 (IL-18), and IL-1*β* were determined by quantitative real-time PCR analysis, western blot, and ELISA analyses as appropriate. Moreover, the expression levels of the PI3K/Akt pathway key proteins were determined by quantitative real-time PCR analysis and western blot assays. To determine the PI3K/Akt pathway involvement in GLGZG-mediated neuroprotection, the PI3K inhibitor LY294002 (LY, 10 *μ*M) was added. The expression levels of NeuN, Akt, and p-Akt were evaluated. *Results*. It was found that GLGZG could inhibit OGD/R-induced cell apoptosis, increase neuronal cell viability, decrease the production of IL-18 and IL-1*β*, and downregulate the expression levels of pyroptosis markers (NLRP3, ASC, and caspase-1). Furthermore, GLGZG could modulate the PI3K/Akt signaling pathway. Pharmacological inhibition of the PI3K pathway not only abrogated the effects of GLGZG on Akt but also neutralized its prosurvival and antipyroptotic actions. *Conclusions*. The findings indicated that GLGZG pretreatment effectively reduced OGD/R-induced injury by inhibiting cell pyroptosis and activating the PI3K/Akt pathway. These data provide important evidence for the therapeutic applications of this regimen in ischemic stroke.

## 1. Introduction

Stroke, notably ischemic stroke, is a life-threatening disease, which is considered the most common cause of disability and mortality in the world [1, 2]. According to worldwide statistics, approximately 75% of the patients who survive face long-term physical and cognitive disabilities. In addition, stroke has significant social and economic consequences [3]. Stroke is mainly classified as ischemic or hemorrhagic with the former accounting for more than 80% of all cases [4]. Ischemic stroke causes cerebral blood flow decline, which may lead to deprivation of brain oxygen and glucose supply, and subsequent induction of apoptosis, necrosis, and metabolic dysfunction in neuronal cells [5]. These effects cause brain damage. Currently, improving the cerebral blood circulation has been the focus of attention. This is achieved by thrombolytic, antiplatelet, and anticoagulation therapies that aim to stimulate vasodilation. Moreover, administration of neuroprotective drugs is frequently used. However, these treatments are limited by a narrow therapeutic time window and secondary side effects [6]. Therefore, novel therapeutic agents, which not only can exert endogenous protection, but also can enhance the resistance of nerve cells to ischemia and hypoxia, are needed.

Ischemic stroke involves the interaction of numerous pathophysiological processes. Accumulating evidence has shown that inflammation and apoptosis are associated with ischemic stroke. Recently, a form of programmed cell death called pyroptosis was observed in cerebral ischemia and reperfusion injury [7]. Pyroptosis is not observed under homeostatic conditions and does not contribute to embryonic development. However, it is closely associated with the inflammatory response [8, 9]. The induction of pyroptosis relies on the inflammasome and caspase-1 activation. The induction of proinflammatory mediators, such as pro-IL-1*β*, NLRP3, and caspase-11, is mediated by transcriptional activation of their corresponding genes that causes assembly of the inflammasome and activation of caspase-1. Active caspase-1 causes proteolytic maturation of pro-IL-1*β* and pro-IL-18 into their active forms and induces pyroptosis. IL-1*β*, caspase-1, and NLRP3 have been reported to play critical roles in pyroptosis [9–12]. Targeting pyroptosis is considered a viable therapeutic strategy to improve the clinical outcomes in patients with ischemic stroke [13–15].

Chinese classical prescriptions are important resources to develop safe and effective candidates for ischemic stroke treatment. The Gualou Guizhi granule (GLGZG, Min drug system approval no. S20130001) is a standard hospital prescription used at the Fujian University of the TCM Affiliated Second People's Hospital (Fuzhou, China), which was initially reported by Zhang Zhongjing in JinKui Yao Lue [16]. It consists of the following six types of Chinese herbs: *Trichosanthes kirilowii* Maxim., *Paeonia lactiflora* Pall., *Cinnamomum cassia* Presl., *Glycyrrhiza uralensis* Fisch., *Zingiber officinale* Rosc., and *Ziziphus jujuba* Mill. It has long been used in the clinic to treat muscular spasticity and dyskinesia following stroke, epilepsy, or spinal cord injury in China with a weight ratio of 10 : 3:3 : 3:2 : 3 [17–22]. Recent studies have also documented that GLGZG exerts anti-inflammatory, antiapoptotic, and neuroprotective effects in vivo and in vitro [23–26]. Our group has shown that GLGZG suppresses LPS-stimulated proinflammatory responses [27, 28]. However, the detailed mechanisms underlying its effects remain unknown. In the present study, the oxygen-glucose deprivation and reoxygenation (OGD/R) of primary neurons were used in an in vitro model of I/R injury to evaluate the protective effects of GLGZG and the possible signaling pathways involved.

## 2. Materials

GLGZG was provided by the Pharmaceutical Department of Fujian University of the Traditional Chinese Medicine Affiliated Second People's Hospital (Fuzhou, China). It was certified and standardized on the basis of labeled compounds (The Food and Drug Administration in the Fujian Province 2013). It was deposited in the school of pharmacy of the People's Republic of China at the Fujian University of Traditional Chinese Medicine. GLGZG was dissolved in medium at 1mg/ml before use. IL-1*β*, IL-6, and TNF-*α* were measured by ELISA kits (ABclonal Biotechnology Co., Ltd., Wuhan, China). Rabbit antibodies against Akt, p-Akt (Ser473), PI3K (p85), PI3K (p110*α*), IKK*β*, p-I*κ*B*α*, I*κ*B*α*, NF-*κ*Bp65, and *β*-actin were purchased from Cell Signaling Technology (Boston, MA, USA). The secondary antibodies conjugated with horseradish peroxidase (HRP) were all purchased from Xiamen Lulong Biotech Co., Ltd. (Xiamen, China). The PI3K inhibitor LY294002 (LY) was purchased from Sigma (St. Louis, MO, USA).

## 3. Methods

### 3.1. UPLC-MS/MS Analysis of GLGZG

An ultraperformance liquid chromatography-triple quadrupole mass spectrometry (UPLC-MS/MS) method was used to analyze GZGZG based on our previous study [29, 30]. Chromatographic separation was carried out by a Waters ACQUITY UPLC system (Waters, Milford, MA, USA), and tandem mass spectrometry was performed on a Waters Xevo TQMS (Milford, MA, USA) with an electrospray ion source (ESI). All compounds were detected in a negative ion mode.

### 3.2. Primary Neuron Culture

Primary neurons were isolated from the cerebral hippocampi of newborn SD rats (from the Center of Experimental Animal, Fujian University of Traditional Chinese Medicine) within 24 h and were prepared and verified according to the protocol previously described [25]. Rats were deeply anesthetized and sacrificed by cervical dislocation. Briefly, following removal of the meninges, the hippocampi were collected and digested in papain (Sigma) for 15 min at 37°C. The cells were dissociated by pipetting up and down. The cells were seeded on poly-D-lysine (Sigma) coated 96-well plates or 6-well plates with serum-free Neurobasal medium (Life Technologies) supplemented with 2% B-27 serum-free supplement (Life Technologies) and 0.5 mM GlutaMAX™-I (Life Technologies).The cells were incubated at 37°C in a humidified atmosphere of 5% CO_2_ atmosphere. Approximately half of the culture medium was replaced every third day.

### 3.3. Oxygen-Glucose Deprivation and Reperfusion (OGD/R)

Primary neurons were exposed to OGD conditions and were cultured in glucose-free Neurobasal medium in an anaerobic chamber (Thermo Fisher Scientific) filled with 1% O_2_, 5% CO_2_, and 94% N_2_ for 90 min. Following OGD, primary neurons were cultured in glucose-containing medium under standard conditions of 95% O_2_ and 5% CO_2_ for 20 h. The condition was determined comprehensively considering use in the preliminary experiment.

### 3.4. GLGZG Treatment

Working GLGZG dilutions (100, 300, and 500 *μ*g/ml, which was determined comprehensively considering use in the preliminary experiment) in serum-free Neurobasal medium supplemented with 2% B-27 serum-free medium and 0.5mM GlutaMAX™-I were prepared freshly and used to pretreat cultures for 24 h prior to exposure to OGD. These conditions were maintained throughout the duration of the experiment. Control cells were incubated with an equal amount of medium. The effects of the PI3K inhibitor LY294002 (LY) 25 *μ*M 30 min prior to GLGZG intervention were also investigated.

### 3.5. MTT Assay

The cell viability was assessed by the 3-(4,5-dimethylthiazol-2-yl)-2, 5-diphenyltetrazoliumbromide (MTT, Sigma, St. Louis, MO, USA) assay. The incubation and treatment of primary neurons were carried out according to the experimental requirements. MTT (10 *μ*l) was added and incubated for an additional 4 h at 37°C. Afterwards, the medium was removed, and the dye crystals were dissolved in 100 *μ*l DMSO. Subsequently, the absorbance was measured at 570 nm by a microplate reader (Infinite M200 Pro, TECAN). The cell viability was presented as the percentage of the average absorbance compared with the control group.

### 3.6. Assessment of Hoechst 33342 Staining

Cell death was assessed by Hoechst 33342 staining. The incubation and treatment of primary neurons were carried out according to the experimental requirements. The neurons were washed with precooled PBS and fixed with 4% paraformaldehyde for 15 min at room temperature, followed by washing with PBS for 5 min three times. The cells were blocked with PBS containing 10% goat serum and 0.3% Triton X-100 for 1 h at room temperature and subsequently incubated with Hoechst 33342 for 5 min. Following staining, the neurons were monitored using an inverted fluorescence microscope.

### 3.7. ELISA

The incubation and treatment of primary neurons were carried out according to the experimental requirements. The supernatant samples were collected, and IL-1*β* and IL-18 levels were measured by ELISA (RayBiotech, Atlanta, USA) according to the manufacturers' instructions.

### 3.8. Immunofluorescence Assay

The immunofluorescence assay was performed to detect the characteristic indicator of neurons, NSE. In brief, cells were fixed using 4% paraformaldehyde for 15 min at room temperature, followed by washing with PBS three times for 5 min. The cells were blocked with 5% BSA prepared in 0.1% Tween 20 (PBST) and 0.3% Triton X-100 for 1 h at room temperature. Subsequently, the cells were incubated with NSE specific primary antibody (1 : 500, Thermo Fisher Scientific, Waltham MA, USA) overnight at 4°C. Following washing, the cells were incubated with FITC-labeled IgG secondary antibody (Beijing Zhongshan Jinqiao Biotechnology Co., Ltd., Beijing, China; 1 : 200 dilution in 5% BSA solution) for 1 h in the dark. Subsequently, the cells were stained with 25 *μ*g/ml 4'-6-diamidino-2-phenylindole (DAPI) in PBST. Finally, the samples were documented using Olympus IX73 fluorescence microscope (Tokyo, Japan).

### 3.9. Quantitative Real-Time PCR Analysis

Total RNA was extracted by RNeasy® Mini Kit (QIAGEN, Hilden, Germany), and the first strand cDNA was synthesized using the RevertAid First Strand cDNA Synthesis Kit (Thermo Fisher Scientific). Quantitative PCR amplification was performed using SYBR Green Master Mix (Roche Life Science, USA) on an ABI 7900HT real-time PCR system (Applied Biosystems, Inc., Foster City, CA, USA). The data were analyzed by the 2^−ΔΔCT^ relative quantification method. The relative transcriptional levels of the target genes were normalized to those of GAPDH. The primer sequences for the amplification of the target genes are shown in Table 1. The relative transcriptional levels of the target genes were normalized to those of GAPDH and were calculated.

### 3.10. Western Blot Analysis

The incubation and treatment of primary neurons were carried out according to the experimental requirements. The cells were collected and lyzed using lysis buffer. The cells were centrifuged at 12,000 g for 15 min. The supernatant was collected, and the protein concentration was determined by the BCA method. The protein was mixed with loading buffer and incubated at 100°C for 6 min. Ultimately, the samples were probed for western blot analysis with primary antibodies to NLRP3 (1 : 1000), caspase-1 (1 : 1000), ASC (1 : 1000), Bax (1 : 1000), Bcl-2 (1 : 1000), NeuN (1 : 1000), p-Akt (Ser473) (1 : 2000), Akt (1 : 1000), PI3K (p85) (1 : 1000), PI3K (p110*α*) (1 : 1000), PDK1(1 : 1000), p-PDK1(1 : 1000), PTEN (1 : 1000), and *β*-actin (1 : 1000) overnight at 4°C . Finally, they were evaluated using the ECL western detection reagents, and the relative expression levels of the target genes that were normalized to *β*-actin expression levels were analyzed.

### 3.11. Statistical Analysis

All data were statistically analyzed by the SPSS 22.0 software and expressed as mean ± standard deviation (`*x* ± s). The difference between groups was analyzed by one-way ANOVA, and a *P* < 0.05 was considered for significant differences.

## 4. Results

### 4.1. UPLC-MS/MS Analysis of GLGZG

**Figure 1:**
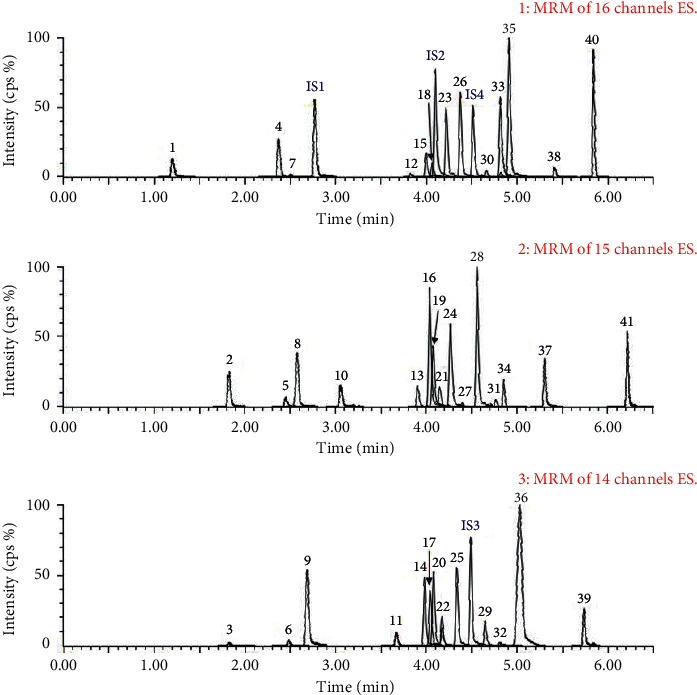
The MRM chromatograms of 41 compounds present in GLGZG: (1) gallic acid, (2) protocatechuic acid, (3) neochlorogenic acid, (4) oxypaeoniflorin, (5) chlorogenic acid, (6) catechin, (7) protocatechuic aldehyde, (8) p-hydroxybenzoic acid, (9) methyl gallate, (10) vanillic acid, (11) albiflorin, (12) schaftoside, (13) paeoniflorin, (14) 4-hydroxycinnamic acid, (15) rutin, (16) ethyl gallate, (17) liquiritin apioside, (18) pentagalloylglucose, (19) liquiritin, (20) luteoloside, (21) ferulic acid, (22) astragalin, (23) 3-hydroxycinnamic acid, (24) isoliquiritin apioside, (25) isoliquiritin, (26) 2-hydroxycinnamic acid, (27) ononin, (28) liquiritigenin, (29) benzoylpaeoniflorin, (30) jujuboside (A), (31) cinnamic acid, (32) formononetin, (33) jujuboside (B), (34) 2-methoxycinnamic acid, (35) isoliquiritigenin, (36) glycyrrhizic acid, (37) 6-gingerol, (38) licochalcone (A) (39) 8-gingerol, (40) 6-shogaol, and (41) glycyrrhetinic acid.

As shown in Figure 1, 41 major target components of GLGZG were identified by UPLC-MS/MS. The components were as follows: (1) gallic acid, (2) protocatechuic acid, (3) neochlorogenic acid, (4) oxypaeoniflorin, (5) chlorogenic acid, (6) catechin, (7) protocatechuic aldehyde, (8) p-hydroxybenzoic acid, (9) methyl gallate, (10) vanillic acid, (11) albiflorin, (12) schaftoside, (13) paeoniflorin, (14) 4-hydroxycinnamic acid, (15) rutin, (16) ethyl gallate, (17) liquiritin apioside, (18) pentagalloylglucose, (19) liquiritin, (20) luteoloside, (21) ferulic acid, (22) astragalin, (23) 3-hydroxycinnamic acid, (24) isoliquiritin apioside, (25) isoliquiritin, (26) 2-hydroxycinnamic acid, (27) ononin, (28) liquiritigenin, (29) benzoyl paeoniflorin, (30) jujuboside A, (31) cinnamic acid, (32) formononetin, (33) jujuboside B, (34) 2-methoxycinnamic acid, (35) isoliquiritigenin, (36) glycyrrhizic acid, (37) 6-gingerol, (38) licochalcone A, (39) 8-gingerol, (40) 6-shogaol, and (41) glycyrrhetinic acid in GLGZG which were identified and quantified by UPLC-MS/MS. The total contents of the investigated 41 components in GLGZG were estimated to be 15.5 mg/g.

### 4.2. GLGZG Protects against OGD/R Injury

**Figure 2:**
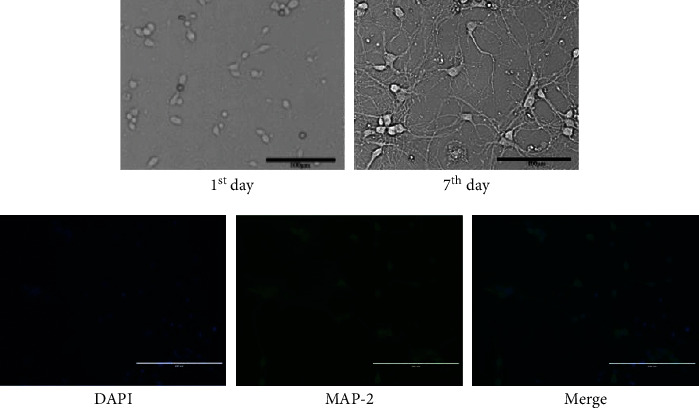
Morphology of cultured neurons and immunofluorescent staining of neurons with MAP-2 and DAPI. Green fluorescence indicates MAP-2-positive neurons; blue fluorescence indicates nuclei of total neurons.

**Figure 3:**
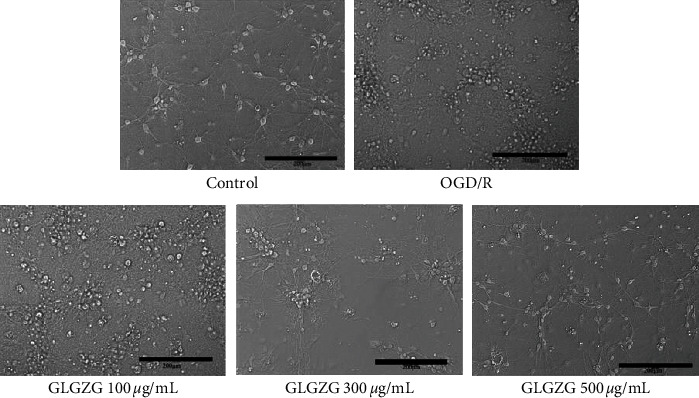
Effects of Gualou Guizhi granule (GLGZG) on the changes caused by OGD/R-induced morphological characteristics. Representative bright field microscopy images indicate the morphological differences among control, OGD/R, and OGD/R + GLGZG-treated (100, 300, and 500 *μ*g/ml) neurons.

The morphology of the cultured neurons is shown in Figure 2. The neurons were round-shaped with abundant dendrites and extended protrusions. They were able to form a neural network on day seven. Immunofluorescent staining demonstrated that cell bodies and neurites of neurons were stained green with MAP-2 and that their nuclei were labeled blue with DAPI. The results suggested that approximately 90% of the cells were neurons. As shown in Figure 3, following the treatment of GLGZG and OGD/R, the morphological examination results indicated that the number of neurons was reduced and the dendrites disappeared. GLGZG pretreatment could protect from neuron injury induced by OCD/R.

**Figure 4:**
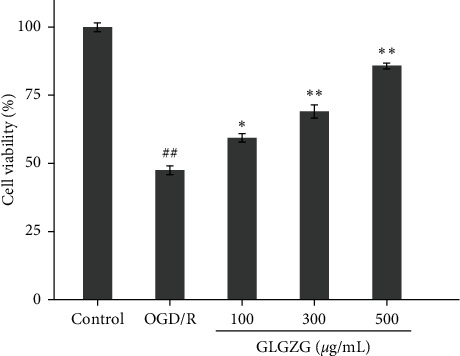
The effects of the Gualou Guizhi granule (GLGZG) on OGD/R-induced neuronal cell death. Gualou Guizhi granule (GLGZG) suppressed OGD/R-induced loss of cell viability. Cell viability was measured using the MTT assay (*n* = 6). The results were expressed as percentages relative to the control group. The data were shown as mean ± SD of six independent experiments. The experiments were performed in triplicate. ^*##*^*P* < 0.01 vs. control group; ^*∗*^*P* < 0.05, ^*∗∗*^*P* < 0.01 vs. OGD/R group.

The survival rate of neurons was significantly reduced to 47.6 ± 1.2% following OGD/R compared with that noted in the control group (*P* < 0.01). GLGZG pretreatment can increase cell viability in neurons. The cell viability was estimated to be 59.4 ± 1.9%, 69.2 ± 2.4% and 85.9 ± 0.9% (*P* < 0.05 and *P* < 0.01; Figure 4) for the three different groups, respectively.

**Figure 5:**
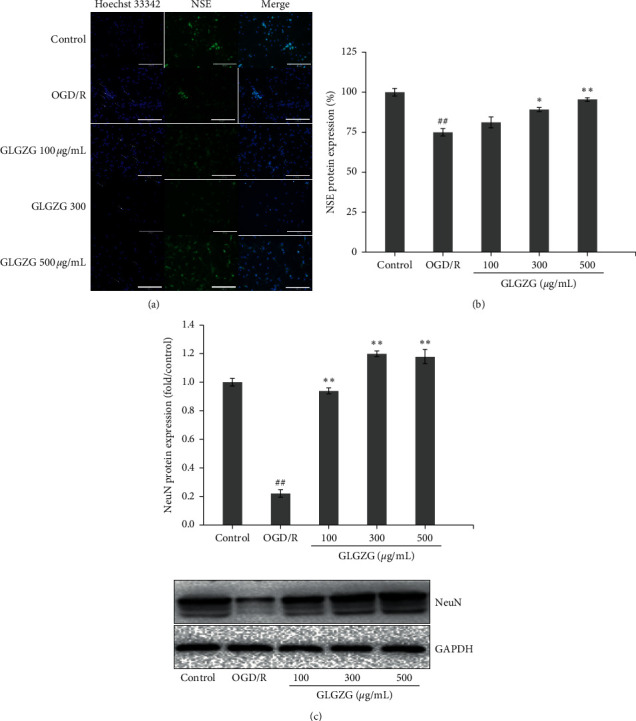
Effects of the Gualou Guizhi granule (GLGZG) on NeuN and NSE protein expressions in neurons following OGD/R. (a) Representative photomicrographs of NSE were determined by IF. Green fluorescence corresponds to NSE-positive neurons; blue fluorescence is representative of the total number of nuclei in neurons. (b) The percentage of NSE-positive neurons was estimated by dividing the number of NSE-positive neurons with the total number of neurons in five high-power fields. (c) Western blot and quantitative analyses of NeuN protein levels. The data were shown as means ± SD. ^*##*^*P* < 0.01 vs. control group; ^*∗*^*P* < 0.05, ^*∗∗*^*P* < 0.01 vs. OGD/R group.

In addition, NeuN and NSE protein expression levels were evaluated in order to further confirm that GLGZG protected against OGD/R injury (Figure 5). The results indicated that NeuN and NSE protein expression levels were significantly decreased in OGR/R compared with those noted in the control group (*P* < 0.01). GLGZG pretreatment reversed this decrease. The data indicated that GLGZG could inhibit apoptosis induced by OGD/R injury.

### 4.3. GLGZG Attenuated OGD/R-Induced Pyroptosis

**Figure 6:**
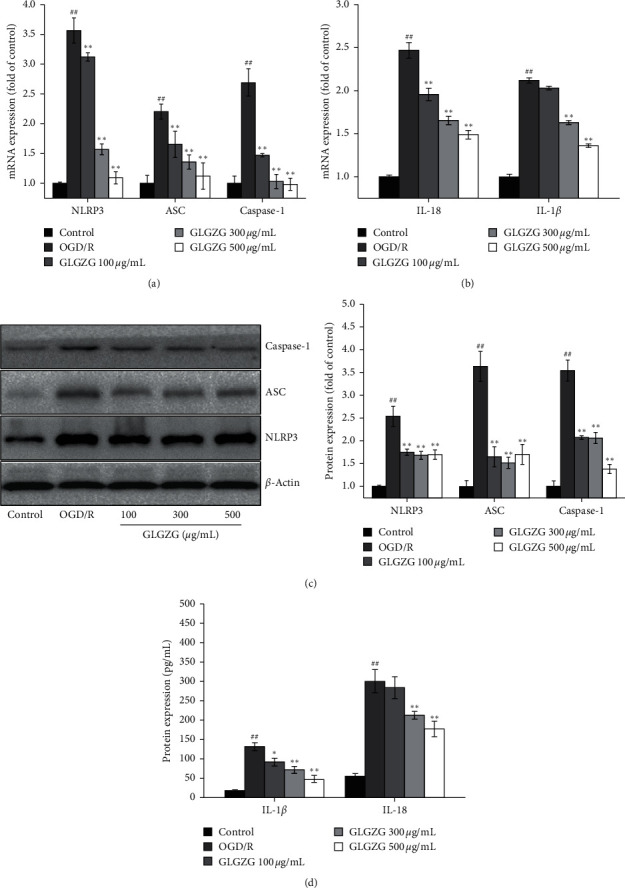
Effects of Gualou Guizhi granule (GLGZG) on expression of pyroptosis markers in neurons following OGD/R. (a, b) NLRP3, ASC, caspase-1, IL-18, and IL-1*β* mRNA levels. (c) Representative western blots and relative density of NLRP3, ASC, and caspase-1, and (d) IL-18 and IL-1*β* levels. The data are shown as mean ± SD. ^##^*P* < 0.01 vs. control group; ^*∗*^*P* < 0.05, ^*∗∗*^*P* < 0.01 vs. OGD/R group.

Our previous studies have shown that GLGZG could suppress neuronal apoptosis [25]. In order to determine whether GLGZG specifically targeted OGD/R-induced pyroptosis, the expression levels of pyroptosis markers were analyzed. As shown in Figures 6(a) and 6(b), the mRNA expression levels of NLRP3, ASC, caspase-1, IL-18, and IL-1*β* were markedly increased following OGD/R compared with those of the control group. GLGZG pretreatment could decrease the levels of the aforementioned markers. Similarly, the protein expression of NLRP3, ASC, and caspase-1 (Figures 6(c) and 6(d)) and the content of IL-18 and IL-1*β* (Figure 6(d)) were significantly higher than those noted in the neurons of the OGD/R group. These effects were decreased following GLGZG treatment.

### 4.4. GLGZG Inhibited OGD/R-Induced Pyroptosis by Modulating PI3K/Akt Signaling

**Figure 7:**
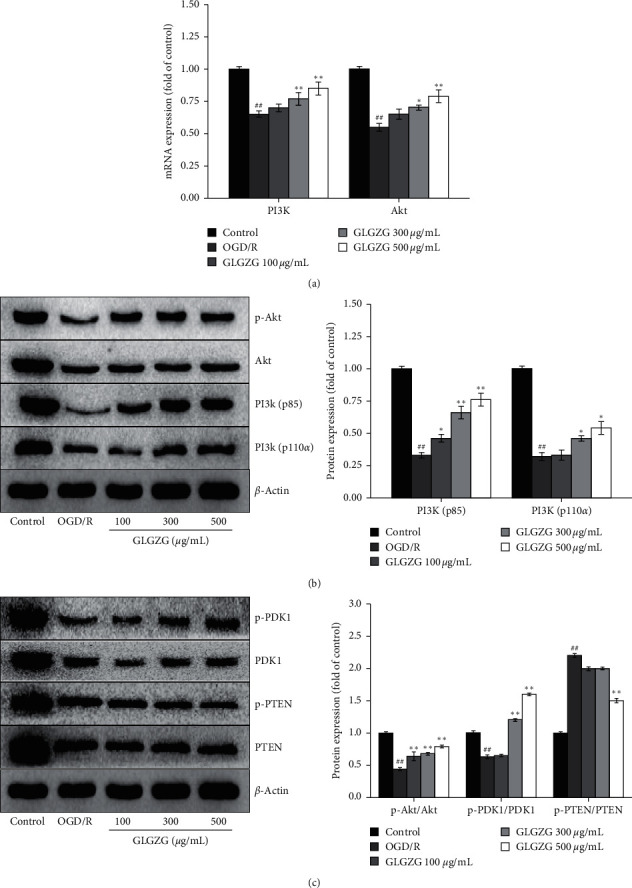
The effects of the Gualou Guizhi granule (GLGZG) on PI3K/Akt signaling in neurons following OGD/R. (a) PI3K and Akt mRNA levels (b, c). Representative western blots and relative density of PI3K, Akt, PDK1, and PTEN levels. The data were shown as means ± SD. ^##^*P* < 0.01 vs. control group; ^*∗*^*P* < 0.05, ^*∗∗*^*P* < 0.01 vs. OGD/R group.

As shown in Figure 7(a), following OGD/R, PI3K and Akt mRNA levels were markedly decreased compared with those of the control group and were restored to normal levels by GLGZG treatment. The results were verified by western immunoblotting (Figures 7(b) and 7(c)). Moreover, PI3K/Akt protein levels were assessed. The results of the western blot analysis indicated that the expression levels of p-Akt/Akt and p-PDK1/PDK1 in the OGD/R group were drastically decreased, whereas the levels of p-PTEN/PTEN were apparently increased. The expression levels of the p-Akt/Akt and p-PDK1/PDK1 proteins were increased (*P* < 0.01), whereas the expression levels of the p-PTEN/PTEN proteins were decreased (*P* < 0.01) in the GLGZG group.

**Figure 8:**
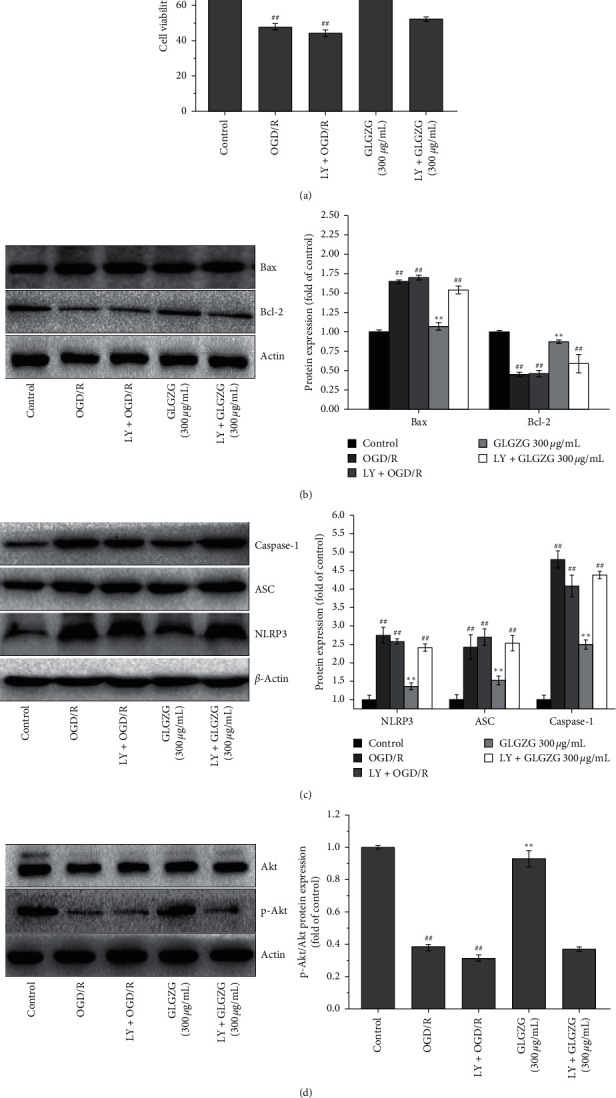
Pharmacological inhibition of PI3K abrogated the neuroprotective effects of the Gualou Guizhi granule (GLGZG) against OGD/R insult. LY294002 reversed GLGZG-mediated (a, b) neuronal survival post-OGD/R. (c) Downregulation of pyroptosis markers and (d) Akt activation. The data were shown as mean ± SD. ^##^*P* < 0.01 vs. control group; ^*∗*^*P* < 0.05, ^*∗∗*^*P* < 0.01 vs. OGD/R group.

To further confirm whether GLGZG protects against OGD/R-induced injury by inhibiting cell pyroptosis via the PI3K/Akt signaling pathway, the specific PI3K inhibitor LY294002 was employed to block PI3 kinase-dependent Akt phosphorylation and kinase activity. The results of the present study demonstrated that PI3K inhibition 1 h prior to GLGZG not only abrogated its prosurvival (Figure 8(a)) and antipyroptotic (Figure 8(b)) ability but also failed to restore the levels of the pyroptosis markers (Figure 8(c)). Furthermore, GLGZG also failed to promote the phosphorylation of Akt (Figure 8(d)). These data suggested that GLGZG protected neuronal cells against OGD/R-induced injury by inhibiting cell pyroptosis via the PI3K/Akt signaling pathway.

## 5. Discussion

Neuroinflammation is a vital hallmark of ischemic stroke pathology, which involves a cascade of inflammatory reactions. In addition, recent studies have shown that the inflammasome is assembled, and caspase-1 is activated during that process. Active caspase-1 causes the proteolytic cleavage of pro-IL-1*β* and pro-IL-18 into their active forms and induces pyroptosis, a proinflammatory form of regulated cell death. In parallel, pyroptosis releases proinflammatory cytokines and danger signals [9]. The targeting of pyroptosis has gained considerable attention and may be considered a new therapeutic strategy for inflammation caused by cerebral ischemia and reperfusion injury.

In recent years, Gualou Guizhi granule (GLGZG, Min drug system approval no. S20130001), a standard hospital prescription at Fujian University of the TCM Affiliated Second People's Hospital (Fuzhou, China), is widely applied in the clinic to treat muscular spasticity and dyskinesia following stroke, epilepsy, or spinal cord injury in China [18–22]. Recent studies have shown that GLGZG can attenuate cerebral ischemia reperfusion-induced brain injury and neurological deficit [23–26], which protects against pentetrazol-induced epilepsy [31]. All these studies suggested that GLGZG may exert neuroprotective effects on cerebral ischemia reperfusion injury. In the current study, it was found that GLGZG pretreatment at doses of 100, 300, and 500 *μ*g/ml could attenuate OGD/R injury as demonstrated by increased cellular viability and decreased neuronal apoptosis.

Accumulating evidence has shown that neuroinflammation plays an important role in the pathological process involved in ischemic stroke [32]. The levels of the proinflammatory cytokines, tumor necrosis factor (TNF-*α*), IL-1*β*, and IL-6 were dramatically elevated. Our group demonstrated that GLGZG could inhibit high TNF-*α*, IL-1*β*, and IL-6 levels, as well as the levels of monocyte chemotactic protein 1 (MCP-1) and nitric oxide (NO) in MCAO-induced rats [27]. Furthermore, GLGZG has also been shown to reduce generation of TNF-*α*, NO, IL-6, IL-1*β*, and IL-8 in models of LPS-induced microglia (BV2) activation [27, 28]. GLGZG exhibited significant anti-inflammatory effects. In the present study, GLGZG inhibited the high production of IL-1*β* and IL-18 in OGD/R-induced neurons. IL-1*β*, caspase-1, and inflammasomes have been reported to play important roles in cerebral ischemia reperfusion injury [7]. The inflammasome is assembled and caspase-1 is activated, which subsequently induces secretion of proinflammatory cytokines, such as IL-1*β* and IL-18, as well as a form of cell death called pyroptosis. In the present study, real-time PCR, ELISA, and western blot analyses demonstrated that the increase in the levels of IL-1*β* and caspase-1 and NLRP3 inflammasome was significantly prevented by GLGZG pretreatment of the cells. Moreover, GLGZG further downregulated ASC, which acted as the adaptor protein connecting the PRRs and procaspase-1 [33].

The PI3K/Akt pathway is one of the major signaling pathways that have been identified as important players in regulating cell proliferation, growth, survival, and angiogenesis. Activation of the PI3K/Akt pathway has been proven to decrease the induction of inflammatory genes and is also influenced by the NLRP3 inflammasome [34]. In the present study, the results indicated that GLGZG could activate PI3K (p85), PI3K (P110*α*), p-Akt (Ser473), and p-PDK1 in OGD/R-induced neurons. In addition, to further define whether GLGZG protected against OGD/R-induced injury by inhibiting cell pyroptosis via the PI3K/Akt signaling pathway, the specific PI3K inhibitor LY294002 was applied. Pharmacological inhibition of PI3K not only abrogated the effects of GLGZG on Akt but also neutralized its prosurvival and antipyroptotic action.

GLGZG is a complex Chinese herbal prescription, as demonstrated by our UPLC-MS/MS analysis and previous phytochemical studies [29, 30, 35]. A total of 104 different compounds were identified in GLGZG, and several bioactive components, such as citrulline, luteolin, puerarin, liquiritin, taxifolin, naringin, formononetin, isoliquiritigenin, 6-gingerol, curcumin, caffeic acid, ferulic acid, jujuboside A, protocatechuic acid, cinnamic acid, catechin, and paeoniflorin, were characterized. Previous studies have demonstrated that these compounds, such as Trichosanthes kirilowii Maxim. [36, 37], protocatechuic acid [38], catechinic acid [39], curcumin [40], 6-gingerol [41], paeoniflorin [42], isoliquiritigenin [43], and liquiritin [18], exhibited a protective effect on cerebral ischemia reperfusion injury. Previous studies conducted by our group further demonstrated that paeoniflorin [44–46] exerted neuroprotective effects. In addition, the present study indicated that citrulline, albiflorin, paeoniflorin, liquiritin, liquiritin apioside, isoliquiritin apioside, isoliquiritigenin, and glycyrrhizinic acid could be absorbed into the blood and could penetrate BBB [47]. So, using GLGZG extract on neurons could elucidate something. However, the efficacy of these constituents on cell pyroptosis is not yet fully determined, and further studies are necessary to reveal their mechanism of action.

## 6. Conclusions

The findings of the present study indicated that GLGZG pretreatment effectively reduced OGD/R-induced injury by inhibiting cell pyroptosis. In addition, the PI3K/Akt pathway played a crucial role in the neuroprotective effects of GLGZG. These results provided evidence that GLGZG may be a neuroprotective nutrient against OGD/R, which may be a promising and safe complementary agent against ischemic stroke.

## Figures and Tables

**Table 1:** Primers used for quantitative real-time PCR analysis.

Gene	Forward primer	Reverse primer
*IL-1β*	5′-ATG ACC TGT TCT TTG AGG CTG AC-3′	5′-CGA GAT GCT GCT GTG AGA TTT G-3′
*IL-18*	5′-CAT GCC ATG GCT GCT GAA CCA GTA GAA GA-3′	5′-CGG GAT CCA ATA GCT AGT CTT CGT TTT G-3′
*NLRP3*	5′-CCAGACCTCCAAGACCACGACT-3′	5′-ATCCGCAGCCAATGAACAGA-3′
*Caspase-1*	5′-TGG TCT TGT GAC TTG GAG GA-3′	5′-TGG CTT CTT ATTGGC ACG AT-3′
*ASC*	5′-AGT TTC ACA CCA GCC TGGAA-3′	5′-TTT TCA AGC TGG CTT TTC GT-3′
*PI3K*	5′-CACCACCCAAGCCCACTTCTAT-3′	5′-TTCCTCGCAATAGGTTCTCGGC-3′
*Akt*	5′-GTGGCAAGATGTGTATGAGAAGAAG-3′	5′-GCTGAGTAGGAGAACTGGGGAAAG-3′
*GAPDH*	5′- AGC CCA GAA CAT CAT CCC TG-3′	5′- AGC CCA GAA CAT CAT CCC TG-3′

## Data Availability

The datasets analyzed during the current study are available from the corresponding author on reasonable request.

## Abbreviations

GLGZG: Gualou Guizhi granule
OGD/R: Oxygen-glucose deprivation and reperfusion
NLRP3: NOD-like receptor family pyrin domain-containing 3
ASC: Apoptosis-associated speck-like protein containing a CARD
IL: Interleukin
LY: LY294002.
